# The quantum transition of the two-dimensional Ising spin glass

**DOI:** 10.1038/s41586-024-07647-y

**Published:** 2024-07-10

**Authors:** Massimo Bernaschi, Isidoro González-Adalid Pemartín, Víctor Martín-Mayor, Giorgio Parisi

**Affiliations:** 1 Istituto per le Applicazioni del Calcolo, CNR, Rome, Italy; 2 Departamento de Física Teórica, Universidad Complutense de Madrid, Madrid, Spain; 3 Dipartimento di Fisica, Sapienza Università di Roma, Rome, Italy; 4 Nanotec-Rome unit, CNR, Rome, Italy

**Keywords:** Phase transitions and critical phenomena, Theoretical physics, Statistical physics

## Abstract

Quantum annealers are commercial devices that aim to solve very hard computational problems^1^, typically those involving spin glasses^2,3^. Just as in metallurgic annealing, in which a ferrous metal is slowly cooled^4^, quantum annealers seek good solutions by slowly removing the transverse magnetic field at the lowest possible temperature. Removing the field diminishes the quantum fluctuations but forces the system to traverse the critical point that separates the disordered phase (at large fields) from the spin-glass phase (at small fields). A full understanding of this phase transition is still missing. A debated, crucial question regards the closing of the energy gap separating the ground state from the first excited state. All hopes of achieving an exponential speed-up, compared to classical computers, rest on the assumption that the gap will close algebraically with the number of spins^5–9^. However, renormalization group calculations predict instead that there is an infinite-randomness fixed point^10^. Here we solve this debate through extreme-scale numerical simulations, finding that both parties have grasped parts of the truth. Although the closing of the gap at the critical point is indeed super-algebraic, it remains algebraic if one restricts the symmetry of possible excitations. As this symmetry restriction is experimentally achievable (at least nominally), there is still hope for the quantum annealing paradigm^11–13^.

## Abstract (web summary)

We find that, in the quantum transition of Ising spin glass, the closing of the gap at the critical point can remain algebraic by restricting the symmetry of possible excitations, which is crucial for quantum annealing.

## Main

Optimization problems are ubiquitous in everyday life (think, for instance, of deciding the best delivery route or scheduling jobs for the different tools in a factory). These problems can be mathematically formalized: *N* entities (for example, jobs queuing for their appropriate tools) compete as they try to satisfy their mutually contradictory goals. The overall frustration produced by a particular solution is quantified through a cost function, which one attempts to minimize. This task is best solved with the help of a computer, even for quite small *N*. Research into computational complexity studies how the computational resources (memory, computing time and so on) grow with *N* (ref. ^14^). If, for all known algorithms, the necessary resources grow faster with *N* than any polynomial, for example, like *N*!, the problem is considered hard. A small subset of these problems, named NP-complete, is of particular interest: if an efficient algorithm (with resources scaling polynomially in *N*) were discovered for any of the NP-complete problems, then a vast family of hard optimization problems in this subset would become easy. For physicists, the most familiar example of an NP-complete problem is finding the minimal energy state—the ground state—of an Ising spin-glass Hamiltonian on a non-planar graph^15,16^. This explains the surge of hardware specifically designed for minimizing a spin-glass Hamiltonian through a variety of algorithms and physical principles (see, for example, refs. ^17–22^).

Specifically, the strategy that concerns us here is quantum annealing. Both in the original formulation^11^, and also in its hardware implementation^1,20^, the aim is to solve the situation for spin glasses. In particular, D-wave chips solve Ising spin glass in *D* = 2 spatial dimensions (${D}^{{\prime} } > 2$ can be coded over a D-wave’s *D* = 2 graph^23^; see pages 13 and 49 in ref. ^24^ for more on the definition of *D*).

**Fig. 1:**
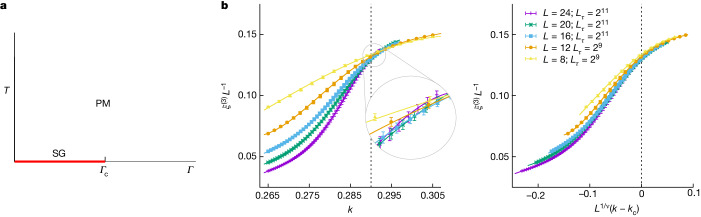
Phase diagram and critical scaling for the two-dimensional quantum spin glass. **a**, Phase diagram for a two-dimensional Ising spin glass in terms of temperature *T* and transverse field *Γ*. For all *T* > 0, the system is disordered when studied at large length scales, so that it is in the paramagnetic phase (PM). At *T* = 0, the ground state seems disordered for *Γ* > *Γ*_c_ (from the point of view of the computational basis). For *Γ* < *Γ*_c_, we encounter the spin-glass phase (SG), which is different for every disorder realization (equation (1)). **b**, Our finite-size scaling analysis (see, for example, refs. ^48,60^) of the critical point at *T* = 0 and *Γ* = *Γ*_c_, in terms of the parameter *k* that represents *Γ* in the Trotter–Suzuki formulation (‘The Trotter–Suzuki formula’ in Methods; *k* grows as *Γ* decreases). Left, correlation length *ξ*^(3)^ in units of the lattice size *L* versus *k*. The curves for the different *L*’s intersect at the critical point *k*_c_ ≈ 0.29. Right, data in the left-hand panel of **b**, when represented as a function of the scaling variable *L*^1/*ν*^(*k* − *k*_c_) with 1/*ν* = 0.7, converge to a limiting curve as *L* grows. Points in **b** are statistical averages, and errors are one standard deviation. Our data set is fully described in Extended Data Table 1. Source Data

Spin glass is the paradigmatic statistical model for quenched disorder^25^. In a transverse field, the Hamiltonian for *S* = 1/2 spins is

1 $$H=-\frac{1}{2}\sum _{x,y}\,\left[\,{J}_{x,y}{\sigma }_{x}^{Z}{\sigma }_{y}^{Z}\right]\,-\,\varGamma \sum _{x}\,{\sigma }_{x}^{X},$$

where *J*_*x,y*_ are the random couplings that define the problem instance under consideration, *Γ* is the transverse field and ${\sigma }_{x}^{X}$ and ${\sigma }_{x}^{Z}$ are, respectively, the first and third Pauli matrices acting on the spin at site *x*. The phase diagram for a two-dimensional interaction matrix *J*_*x,y*_ is sketched in Fig. 1a. For *Γ* = *∞*, in the ground state, all spins are as much aligned with the transverse field as quantum mechanics allows them to be. (Paradoxically, from the point of view of the computational basis that diagonalizes the ${\sigma }_{{\boldsymbol{x}}}^{Z}$ matrices, this ground state seems to be a totally random statistical mixture). As *Γ* is diminished at zero temperature, the ground state varies. In particular, at *Γ* = 0, the ground state encodes the solution of the optimization problem we are interested in. At some point during the annealing, *Γ* goes through the critical value *Γ*_c_ that separates the disordered ground state from the spin-glass ground state, which has a glassy order in the computational basis. This is not just theoretical daydreaming. In a recent experiment conducted on a D-wave chip^23^, some 5,000 qubits displayed coherent quantum dynamics as *Γ* went through *Γ*_c_, for annealings lasting several nanoseconds.

A strong theoretical command of the phase transition at *Γ*_c_ is clearly necessary. A very powerful tool in the analytical study of phase transitions is the renormalization group, which helps to clarify which properties of the critical point are not modified by the different microscopic details of different experiments. Only very broad features, such as symmetries, matter (making it possible to classify problems into universality classes). In fact, the study of disordered systems was one of the early applications of the renormalization group (see, for example, refs. ^26–28^), a strategy that is firmly established for *D* = 2 (ref. ^29^). Yet, it has taken considerable time and effort to show that the renormalization group—and the accompanying universality—also applies to disordered systems for *D* > 2 (refs. ^30–34^; even for *D* = 2, this was a hard endeavour for spin glasses^35^). Unfortunately, the study of the quantum spin-glass transition at finite *D* is considerably behind its thermal counterpart. Essentially, only the *D* = 1 case is well understood^36–39^.

The second-simplest problem to analyse, a spin glass with *D* = 2, poses quite a challenge. Indeed, different approaches have produced mutually contradictory predictions for the crucial physical quantity that ultimately determines whether the quantum computational complexity of the problem to be considered is smaller than its classical counterpart. We are referring to the energy gap *Δ* that separates the ground state from the first excited state of the Hamiltonian (1). Indeed, the required annealing time grows with 1/*Δ*^2^ (ref. ^40^). In a spin glass with *N* ∝ *L*^2^ quantum spins at *Γ* = *Γ*_c_, where *L* is the linear size of the system, *Δ* ∝ *L*^−*z*^, where *z* is the so-called dynamic critical exponent. Early Monte Carlo simulations^5,6,8^ and a series-expansion study^9^ found finite values of *z* (for example, *z* ≈ 1.5 for *D* = 2 spin glasses^5^). A finite *z* is also a crucial assumption of the droplet model for the quantum spin-glass transition^41^. On the other hand, a real-space renormalization group analysis concluded that *z* = *∞* for *D* = 2 or 3 spatial dimensions^10^. (A Monte Carlo simulation too claimed that *z* = *∞* for *D* = 2 (ref. ^42^)).

The starting assumption of refs. ^5,6,8,23^ was a finite value of exponent *z*. Yet, to clarify the aforementioned controversy, our analysis is completely agnostic about *z*. Just as Rieger and Young pushed to the very limit the computational capabilities of the time using special hardware (transputers)^5^, we have performed unprecedented large-scale simulations on graphical processing units (GPUs) using highly tuned custom codes. A careful consideration of the global spin-flip symmetry, implemented by the parity operator $P={\prod }_{x}{\sigma }_{x}^{X}$, turns out to be crucial. Although the gap for same-parity excitations scales algebraically in the number of spins, the gap for parity-changing excitations does close super-algebraically. The coexistence of these two qualitatively different scalings at the critical point is extremely unusual and is probably caused by Griffiths–McCoy singularities^36–39,43^. (In the spin-glass phase, instead, the spontaneously broken parity symmetry naturally generates an exponentially small gap but only for parity-changing excitations.) Although Griffiths–McCoy singularities are strongly dependent on *D*, interestingly, ref. ^44^ hinted at an algebraically scaling gap for same-parity excitations at the quantum critical point of a three-regular graph (a *D* = *∞* problem; parity-changing excitations were not studied in ref. ^44^).

## The ground state

Our aim here is to study the phase transition as seen from the ground state (so that the spectra of excitations and, hence, exponent *z* do not play any role in the analysis in ‘The phase transition’). This entails taking the limit *T* → 0.

In the Trotter formulation that we use (‘The Trotter–Suzuki formula’ in Methods), the original quantum spins on an *L* × *L* lattice are replaced by classical spins on an *L* × *L* × *L*_*τ*_ lattice, *S*_*x*,*τ*_ = ±1. The extra dimension *τ* is named the Euclidean time. *Γ* is replaced by a new parameter *k* that grows as *Γ* decreases. The energy gap *Δ* translates into a correlation length *η* = 1/(*kΔ*) over Euclidean time. In this formulation, the limits *T* → 0 and *L*_*τ*_ → *∞* are equivalent.

Although our main results stem from Monte Carlo simulations, a complementary exact-diagonalization effort on small systems (‘Exact diagonalization’ in the next section and ‘More about exact diagonalization’ in Methods) has been extremely useful, both in shaping our analysis and in providing an understanding for how the limit *T* → 0 is approached (‘At the limit of zero temperature’).

### Exact diagonalization

**Extended Data Fig. 1:**
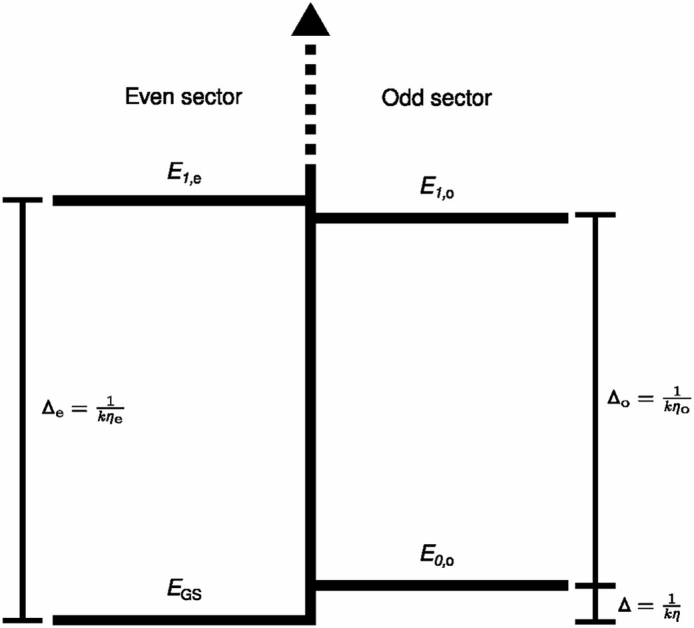
Schematic representation of the energy spectrum. As it is explained in the main text, the parity symmetry splits the spectra into even and odd sectors according to the parity of states. We shall name the even eigenvectors of the transfer matrix (8) as $\left|{0}_{{\rm{e}}}\right\rangle$, $\left|{1}_{{\rm{e}}}\right\rangle$, …, with corresponding eigenvalues ${{\rm{e}}}^{-k{E}_{{\rm{GS}}}}$, and ${{\rm{e}}}^{-k({E}_{{\rm{GS}}}+{\Delta }_{n,{\rm{e}}})}$ for *n* = 1, 2, 3, … [we use the shorthand Δ_e_ = Δ_1,e_]. For the odd sector, we have $\left|{0}_{{\rm{o}}}\right\rangle$, $\left|{1}_{{\rm{o}}}\right\rangle$, … with eigenvalues ${{\rm{e}}}^{-k({E}_{{\rm{GS}}}+\Delta )}$, and ${{\rm{e}}}^{-k({E}_{{\rm{GS}}}+\Delta +{\Delta }_{n,{\rm{o}}})}$ for *n* = 1, 2, 3, … [we use the shorthands Δ = *E*_0,o_ − *E*_GS_, and Δ_o_ = Δ_1,o_]. Notice that expectation values at *T*  =  0 are determined solely by $\left|{0}_{{\rm{e}}}\right\rangle$.

The main lessons that exact diagonalization of systems with size *L* ≤ 6 (‘More about exact diagonalization’ in Methods and Extended Data Figs. 1 and 3) have taught us are the following.

The parity operator *P* splits the spectrum of the Hamiltonian (equation (1)) into even energy levels (*E*_0,e_ < *E*_1,e_ < …) and odd levels (*E*_0,o_ < *E*_1,o_ < …). The ground state is even and its energy is *E*_GS_ = *E*_0,e_.

The first excited state is *E*_0,o_. The minimal gap *Δ* = *E*_0,o_ − *E*_0,e_ displays dramatic fluctuations among samples, up to the point that a statistical analysis should be conducted in terms of log *Δ*. Furthermore, log *Δ* varies notably with *k*. By contrast, the sample-to-sample fluctuations of the same-parity gaps, *Δ*_e_ ≡ *E*_1,e_ − *E*_0,e_ and *Δ*_o_ ≡ *E*_1,o_ − *E*_0,o_ are very mild (also their *k*-dependence is mild). For all our samples, *Δ*_e_ and *Δ*_o_ are of similar magnitude and, unless *Δ* turns out to be inordinately large, *Δ*_e_, *Δ*_o_ ≫ *Δ*.

Thermal expectation values of even operators (operators ${\mathcal{A}}$ such that ${\mathcal{A}}=P\,{\mathcal{A}}P$) reach their *T* = 0 limit for surprisingly small values of *L*_*τ*_. The reasons for this benign behaviour are understood (‘The limit of zero temperature’ in Methods).

### The phase transition

We turn now to Monte Carlo simulations. The standard spin-glass correlation function, when computed for the ground state, is afflicted by a very large anomalous dimension that makes the spin-glass susceptibility *χ*^(2)^ barely divergent at the critical point^5^. We have circumvented this problem by considering instead the correlation matrix *M* (equation (9) in Methods and refs. ^45–47^). From *M*, one can compute not only *χ*^(2)^ but also a better behaved susceptibility *χ*^(3)^. The corresponding correlation length *ξ*^(3)^ is suitable for a standard finite-size scaling study of the phase transition (see, for example, ref. ^48^), which is illustrated in Fig. 1b.

The analysis in ‘The critical point and critical exponents’ in Methods found that for the critical point *k*_c_, the correlation-length exponent *ν* and exponents *γ*^(*n*)^ (${\chi }^{(n)}({k}_{{\rm{c}}})\propto {L}^{{\gamma }^{(n)}/\nu }$; for a discussion see ‘One-time observables’ in Methods):

2 $${k}_{{\rm{c}}}=0.2905(5),\quad \frac{1}{\nu }=0.71(24)(9),$$

3 $$\frac{{\gamma }^{(2)}}{\nu }=0.27(8)(8),\quad \frac{{\gamma }^{(3)}}{\nu }=1.39(23)(11).$$

The first error estimate is statistical whereas the second error accounts for systematic effects. Note that the bound *ν* ≥ 2/*D* (ref. ^49^) is verified and that ${\chi }^{(2)}\propto {L}^{\frac{{\gamma }^{(2)}}{\nu }\approx 0.3}$, indeed, barely diverges^5^.

### At the limit of zero temperature

The naive approach to the limit *T* → 0 would be to study a fixed set of samples for a sequence of growing Euclidean lengths *L*_*τ*_ and to check when the results become independent of *L*_*τ*_ (indeed, *T* > 0 effects die out as ${{\rm{e}}}^{-{L}_{\tau }/\eta }$). However, according to ‘Exact diagonalization’ above, this is just wishful thinking. Indeed, some instances have an inordinately small gap (and, hence, a huge Euclidean correlation length *η*), and so ${{\rm{e}}}^{-{L}_{\tau }/\eta }\approx 1$ for all values of *L*_*τ*_ that we can simulate (one would like ${{\rm{e}}}^{-{L}_{\tau }/\eta }\ll 1$, instead).

**Fig. 2:**
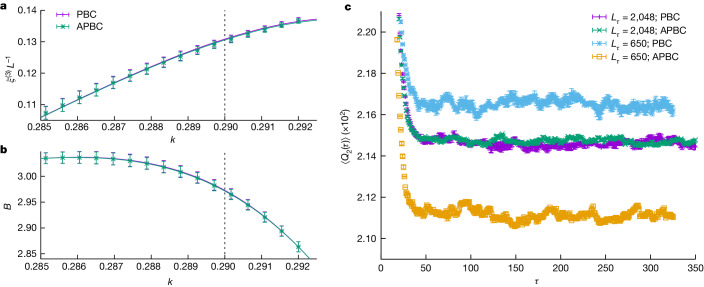
Ensuring that the zero-temperature limit has been reached by comparing PBCs and APBCs over Euclidean time. **a**, Correlation length *ξ*^(3)^ (‘One-time observables’ in Methods) versus *k*, as computed for our largest systems with *L* = 24 and *L*_*τ*_ = 2,048 and with both PBCs and APBCs for the same set of 1,280 samples. The statistical agreement for PBCs and APBCs indicates that the *T* → 0 limit has been effectively reached for this quantity. **b**, As **a** for the Binder cumulant (‘One-time observables’ in Methods). The dashed line represents the critical point, *k*_c_ ≈ 0.29. **c**, The even correlation functions *Q*_2_(*τ*) (‘Two-times observables’ in Methods), as computed for a single sample of *L* = 20 at *k* = 0.29, rather quickly reach their large-*τ* plateau. The functions depend on both *L*_*τ*_ and the boundary conditions. The PBC plateau decreases upon increasing *L*_*τ*_, whereas the APBC plateau notably increases. The reason behind the stronger sensitivity of *L*_*τ*_ for APBCs is understood (‘The limit of zero temperature’ in Methods). Points in **a**, **b**, and **c** are statistical averages, and errors are one standard deviation. Our data set is fully described in Extended Data Table 1. Source Data

Fortunately, considering simultaneously periodic boundary conditions (PBCs) and antiperiodic boundary conditions (APBCs) over Euclidean time offers a way out. A detailed analysis (‘The limit of zero temperature’ in Methods) shows that the sequence of results for growing *L*_*τ*_ converges to *T* = 0 from opposite sides. As *L*_*τ*_ grows (Fig. 2c), the PBC sequence monotonically decreases, whereas the APBC sequence increases. Thus, the statistical compatibility of both types of boundary condition ensures that the zero-temperature limit has been reached (within our statistical errors).

## Spectra of excitations at the transition

The main tool for investigating excitations is the *τ* dependence of the Euclidean correlation function of several operators (‘Two-times observables’ in Methods). It is crucial to distinguish even operators (such as $P\,{\mathcal{A}}P={\mathcal{A}}$) from odd operators ($P\,{\mathcal{A}}P=-{\mathcal{A}}$). For even operators, the decay with *τ* is sensitive only to same-parity gaps (such as *Δ*_e_ and *Δ*_o_ defined in ‘Exact diagonalization’ above). Instead, odd operators feel the different-parity energy gap *Δ*.

For both symmetry sectors, the correlation functions computed for a sample decay exponentially (to zero for odd operators or, Fig. 2c, to a plateau for even operators). In both cases, the correlation lengths *η* and the energy gaps of appropriate symmetry are related as *η* = 1/(*kΔ*). Therefore, what the average over samples of an Euclidean correlation function really features is the probability distribution function (as computed over the different samples) of the correlation lengths *η*.

From now on, we focus on the critical point at *k*_c_ ≈ 0.29. The uncertainty *δk*_c_ about the value of the critical point, approximately 5 × 10^−4^ in our estimate, introduces systematic errors. However, finite-size scaling theory (see, for example, ref. ^48^) tells us that we can quantify these systematic effects by considering the scaling combination $\delta {k}_{{\rm{c}}}{L}_{\max }^{1/\nu }$ where ${L}_{\max }=24$ is the largest lattice size we simulated. This scaling combination corresponds to a value of approximately 0.044. The smallness of this number and the smooth dependence of the scaling functions on the scaling variable $(k-{k}_{{\rm{c}}}){L}^{1/\nu }$ (see, for example, Fig. 1b, right) make it apparent that this source of errors is under control in our simulations. Furthermore, the excellent agreement between our results for same-parity excitations (see below) with a recent experiment (‘Conclusions and outlook’) reinforces this conclusion.

### Even operators

This case is of utmost relevance because only even excited states can cause the system to leave its ground state in (ideal) quantum annealing for the Hamiltonian (equation (1)). Our approach is not entirely satisfying in this respect because, for a given sample, we obtain the smallest of the two same-parity gaps *Δ*_e_ and *Δ*_o_ (one would like to study only *Δ*_e_). Fortunately, both gaps have similar magnitudes (Extended Data Fig. 3).

**Fig. 3:**
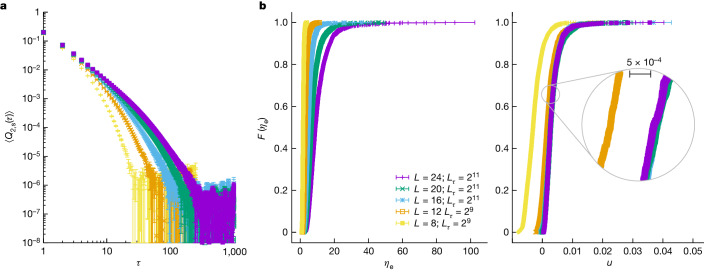
Studying the spectra of even excitations at the critical point. **a**, Sample-averaged subtracted correlation function *Q*_2,s_(*τ*) (‘Fitting process and estimating the Euclidean correlation length’ in Methods) becomes compatible with zero for moderate values of *τ*, for all our system sizes. **b**, Left, after computing the Euclidean correlation length ${\eta }_{{\rm{e}}}^{(s)}$ for each sample, we computed for each *L* the empirical distribution function *F*(*η*_e_), namely the probability *F* of finding a sample with ${\eta }_{{\rm{e}}}^{(s)} < {\eta }_{{\rm{e}}}$ (note the horizontal error bars). Right, the data in the left-hand panel of **b**, when plotted as a function of the scaling variable *u* (equation (4)) do not show any *L* residual *L* dependence other than for our smallest sizes *L* = 8 and 12. Points in **a** and **b** are statistical averages, and errors are one standard deviation. Our data set is fully described in Extended Data Table 1. Source Data

The first optimistic indication comes from the (subtracted) correlation function in Fig. 3a, which goes to zero (within errors) for a moderate value of *τ*. Indeed, the empirical distribution function for the correlation length *η*_e_ in Fig. 3b indicates mild sample-to-sample fluctuations and a relatively weak dependence on *L*. In fact, as shown Fig. 3b, for all *L* > 12, the probability distribution function turns out to depend on the scaling variable

4 $$u=\frac{{\eta }_{{\rm{e}}}-{\eta }_{{\rm{e}}}^{0}}{{L}^{{z}_{{\rm{e}}}}},\quad {\eta }_{{\rm{e}}}^{0}=2.2(3),\quad {z}_{{\rm{e}}}=2.46(17).$$

(Setting ${\eta }_{{\rm{e}}}^{0}=0$, the whole curve cannot be made to scale and the resulting estimate *z*_e_ ≈ 1.7 is lower, see also the concluding paragraph of ‘Two-times observables’ in Methods). Thus, as anticipated, we conclude that the even symmetry sector shows algebraic scaling for its gap.

### Odd operators

**Fig. 4:**
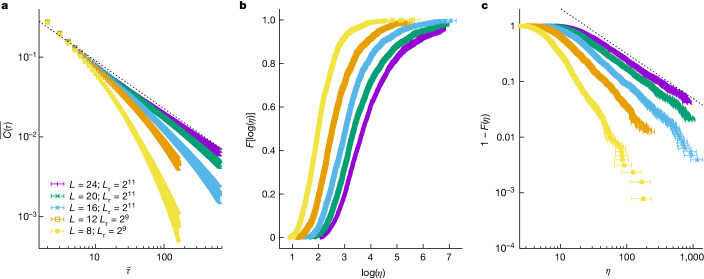
Studying the spectra of odd operators at the critical point. **a**, The decay of the sample-averaged correlation function *C*(*τ*) (equation (14)) approaches a power law as *L* increases. The dashed line is a guide for the eyes. Indeed, we needed to represent *C*(*τ*) in terms of $\widetilde{\tau }=({L}_{\tau }/{\rm{\pi }})\sin ({\rm{\pi }}\tau /{L}_{\tau })$ to avoid distortions due to the PBCs ($\widetilde{\tau }$ and *τ* are almost identical for small *τ*/*L*_*τ*_). **b**, Empirical distribution function *F*(*η*) as a function of $\log \eta$ for all our system sizes. Note that we can compute only up to some *L*-dependent *F* because our largest *L*_*τ*_ is not large enough to allow for a safe determination of *η* in some samples. **c**, For large *η*, the asymptotic behaviour $F(\eta )=1-B/{\eta }^{b}$ is evinced by the linear behaviour (in logarithmic scale) of 1 − *F* as a function of *η*. We fond *b* ≈ 0.8. The dashed line is a guide for the eyes. Points in **a**, **b**, and **c** are statistical averages, and errors are one standard deviation. Our data set is fully described in Extended Data Table 1. Source Data

As would be expected from the exact results for *D* = 1 (refs. ^36,38,39^) and the approximate renormalization group study for *D* = 2 (ref. ^10^), the odd correlation function $\overline{C(\tau )}$ shown in Fig. 4a has, for *L* = 24, a power-law decay $\overline{C(\tau )}\propto 1/{\tau }^{\widetilde{b}}$ with $\widetilde{b}=0.6$. This implies that the magnetic susceptibility—the linear response to a magnetic field aligned with the *z* axis—diverges at the critical point. Indeed, the susceptibility diverges if $\widetilde{b} < 1$ (because it is twice the integral of $\overline{C(\tau )}$ for *τ* going from 0 to *∞*).

Furthermore, $\widetilde{b} < 1$ also for *k* < *k*_c_ (Extended Data Fig. 6). We, therefore, conclude that the susceptibility diverges in the paramagnetic phase. This is exactly the same behaviour encountered for *D* = 1. Accordingly, the probability distribution function of the Euclidean correlation length *η* (recall that *η* = 1/(*kΔ*)) has an extremely fat tail (Fig. 4b,c). This is the behaviour of a Lévy flight, which strongly suggests that the scenario of an infinite-randomness fixed point^10^ is, indeed, realized for *D* = 2 Ising spin glass.

## Conclusions and outlook

We have solved a decades-long controversy through an extreme-scale simulation on GPUs and a careful consideration of the main symmetries of the problem. Our main conclusion is very optimistic: there is no reason in principle why a quantum annealer cannot remain in the ground state while entering the spin-glass phase (Fig. 1a). However, as discussed below, this is not quite the same as solving our optimization problem. To adiabatically enter the spin-glass phase, the annealing time would just need to grow as a power law with the number of quantum spins (equation (4)), provided that parity-changing excitations are avoided (something that, at least nominally, is within the capabilities of current hardware). Let us emphasize that this protective mechanism is crucial for making it possible to study the dynamics at the quantum critical point, as done in recent experiments^23,50^. Entering the spin-glass phase adiabatically is also essential for getting good approximate solutions to optimization problems. Universality and the renormalization group suggest that our optimistic conclusion extends to a vast family of problems (all problems that share the spatial dimensions and the basic symmetries as our spin glass on a square lattice, which includes the interaction graph of D-wave devices).

One may worry that unavoidable experimental effects, for example, tiny but random longitudinal magnetic fields, could break the parity symmetry. Fortunately, the mechanism discussed in the previous paragraph still protects the annealer. Indeed, the gaps internal to the parity sectors, *Δ*_e_ and *Δ*_o_, are similar and much larger than the parity-mixing gap *Δ*. Hence, undesired parity errors will mostly mix the ground states in the even and odd sectors (which are essentially equivalent, as far as the solution to the classical optimization problem is concerned).

We should mention as well that the combination of critical exponents *zν* has been measured in a very recent experiment^50^, which produced results in excellent agreement with those reported in this work.

However, our findings pose, as well, many questions. Let us list a few.

We have seen that entering the spin-glass phase with a quantum annealer should be doable with an effort polynomial in the number of qubits. However, to solve an optimization problem, one still needs to go adiabatically all the way from the critical point to *Γ* = 0. This is a difficult journey, at least for problems with *D* → *∞* spatial dimensions (refs. ^44,51,52^). However, it has been recently announced that an algebraic speed-up, compared to classical algorithms, is within reach^23^. As we said above, having a finite exponent *z* is a basic prerequisite also for an algebraic speed-up.

We know that *D* = 2 Ising glasses may be both hard and easy to solve on a classical computer. For instance, problems formulated for a square lattice with nearest-neighbour interactions can be solved quite efficiently (see, for example, ref. ^53^). However, adding second-neighbour interactions results in an NP-complete problem. As far as we know, it is still unclear whether or not these two problems belong to the same (quantum) computational complexity class. As the second-neighbour interactions should play no role at *Γ*_c_, differences between the two kind of problems (if any) should arise for transverse fields *Γ* < *Γ*_c_.

In this work, we have chosen problem instances with uniform probability, but this is not a necessity. One could focus, instead, on samples that are particularly hard to solve with a classic digital computer^54–56^. It would be interesting to test whether the benign scaling in equation (4) remains unchanged under these challenging circumstances. We know that these classically hard problems are even harder to solve for a D-wave annealer^57^, but there are many possible explanations for this poor performance of quantum hardware (see, for example, refs. ^58,59^).

Another possible avenue of research is concerned with three-dimensional systems. A recent experiment conducted on D-wave hardware suggests *z* ≈ 1.3 (ref. ^23^). Whether this finite dynamic exponent is for only even excitations (as would be the case in two dimensions) or it is unrestricted^6^ is, probably, worthy of investigation.

## Methods

### Model and simulations

Our quantum spins occupy the nodes of a square lattice of side *L*, endowed with PBCs. The coupling matrix *J*_*x*,*y*_ in equation (1) is non-vanishing only for nearest lattice neighbours. A problem instance, or sample, is defined by the choice of the *J*_*x*,*y*_ matrix. The non-vanishing matrix elements, *J*_*x*,*y*_ = *J*_*y*,*x*_ are random, independent variables in our simulations. Specifically, we chose *J*_*x*,*y*_ = ± *J*_0_ with 50% probability. We chose energy units such that *J*_0_ = 1.

Given an observable ${\mathcal{A}}$, we shall refer to its thermal expectation value in a given sample as $\langle {\mathcal{A}}\rangle$ (see equation (8) below; the temperature is as low as possible, ideally *T* = 0). Thermal expectation values are averaged over the choice of couplings (quenched disorder; see, for example, ref. ^25^). We shall denote the second average (over disorder) as $\overline{\langle {\mathcal{A}}\rangle }$.

#### Crucial symmetries

The most prominent symmetries in this problem are the gauge and the parity symmetries. Both symmetries are exact for the Hamiltonian (equation (1)) and for its Trotter–Suzuki approximation (‘The Trotter–Suzuki formula’).

The parity symmetry $P=\prod _{x}{\sigma }_{x}^{X}$ is a self-adjoint, unitary operator that commutes with the Hamiltonian (equation (1)), as well as with the exact (equation (7)) and approximate (equation (8)) transfer matrices. The Hilbert space is divided into two subspaces of the same dimension according to the parity eigenvalues, which are either +1 (even states) or −1 (odd states). We also classify operators as either even ($P\,{\mathcal{A}}P={\mathcal{A}}$) or odd ($P\,{\mathcal{A}}P=-{\mathcal{A}}$). Matrix elements of even operators are non-vanishing only if the two states have the same parity (in contrast, the parity of the states should differ for odd operators). An oversimplified but enlightening cartoon of the spectra in our problem is represented in Extended Data Fig. 1 (see below some exact-diagonalization results that support this view).

Parity symmetry is just a particular type of gauge transformation. Let us arbitrarily choose for each site *n*_**x**_ = 0 or 1. The corresponding gauge operator ${G}_{\{{n}_{x}\}}=\prod _{x}{({\sigma }_{x}^{X})}^{{n}_{x}}$ is self-adjoint and unitary. It transforms the Hamiltonian in equation (1) into a Hamiltonian of the same type but with modified couplings^61^: ${J}_{x,y}\to {J}_{x,y}{(-1)}^{{n}_{x}+{n}_{y}}.$ The gauge symmetry is enforced by the process of taking the disorder average. Indeed, the gauge-transformed coupling matrix has the same probability as the original one. Hence, meaningful observables should be invariant under an arbitrary gauge transformation. The parity operator is obtained by setting *n*_*x*_ = 1 for all sites, which does not modify *J*_*x*,*y*_ (hence, parity is a symmetry for a given problem instance not just a symmetry induced by the disorder average).

#### The Trotter–Suzuki formula

We follow the Trotter–Suzuki approximation^62,63^, which replaces the original quantum spins on an *L* × *L* lattice by classical spins on an *L* × *L* × *L*_*τ*_ lattice, *S*_*x*,*τ*_ = ±1. The extra dimension *τ* is named Euclidean time. We shall write **S** as a shorthand for the *L*^*D*^*L*_*τ*_ spins in the system (*D* = 2, here). Instead, **S**_*τ*_ will refer to the *L*^*D*^ spins at time *τ*. We take equal-strength couplings along the time and space directions (see, for example, ref. ^5^). The probability of **S** is given by

5 $$p({\bf{S}})=\frac{{{\rm{e}}}^{-k{\mathcal{E}}({\bf{S}})}}{Z},\quad Z=\sum _{\{{\bf{S}}\}}{{\rm{e}}}^{-k{\mathcal{E}}({\bf{S}})},$$

where *Z* is the partition function, and

6 $$\begin{array}{l}{\mathcal{E}}({\bf{S}})\,=\,-\mathop{\sum }\limits_{\tau =0}^{{L}_{\tau -1}}\left[\frac{1}{2}\sum _{x,y}\,{J}_{x,y}{S}_{x,\tau }{S}_{y,\tau }+\sum _{x}{S}_{x,\tau }{S}_{x,\tau +1}\right],\\ \,\,\,\,\varGamma \,=\,-\frac{1}{2k}\log \,\tanh \,k.\end{array}$$

Although we have not done so, note that one could use a different coupling over Euclidean time, *k*_*τ*_, which would relate *k* and *Γ* as $\varGamma =-\frac{1}{2k}\log \,\tanh \,{k}_{\tau }$ or, equivalently, ${k}_{\tau }=-\frac{1}{2}\log \,\tanh (k\varGamma )$ (the continuum-time limit at *T* = 0 would be reached by taking *L*_*τ*_ → *∞* first and, afterwards, *k* → 0). PBCs are assumed over Euclidean time. Below, we shall find it useful to consider as well APBCs along only the *τ* direction. Besides, as the reader may check, *k* is a monotonically decreasing function of *Γ*.

Possibly the most direct connection between the *D* + 1 classical spin system and the original quantum problem is provided by the transfer matrix $\widetilde{{\mathcal{T}}}$ (refs. ^64,65^). Let us define ${{\mathcal{H}}}_{0}=-\frac{1}{2}\sum _{x,y}{J}_{x,y}{\sigma }_{x}^{Z}{\sigma }_{y}^{Z}$ and ${{\mathcal{H}}}_{1}=-\varGamma \sum _{x}{\sigma }_{x}^{X}$. The quantum thermal expectation value at temperature *T* = 1/(*kL*_*τ*_) is

7 $$\langle \langle {\mathcal{A}}\rangle \rangle =\frac{{\rm{Tr}}{\mathcal{A}}\,{\widetilde{{\mathcal{T}}}}^{{L}_{\tau }}}{{\rm{Tr}}{\widetilde{{\mathcal{T}}}}^{{L}_{\tau }}},\quad \widetilde{{\mathcal{T}}}={e}^{-k({{\mathcal{H}}}_{0}+{{\mathcal{H}}}_{1})}.$$

Now, for ${\mathcal{A}}={A}_{{\rm{cl}}}(\{{\sigma }_{{\bf{x}}}^{Z}\})$, ${A}_{{\rm{cl}}}$ being an arbitrary function, the Trotter–Suzuki approximation amounts to substituting the true transfer matrix in equation (7) by its proxy ${\mathcal{T}}$ (${\mathcal{T}}=\widetilde{{\mathcal{T}}}+{\mathcal{O}}({k}^{3})$):

8 $$\langle {\mathcal{A}}\rangle =\frac{{\rm{Tr}}{\mathcal{A}}{{\mathcal{T}}}^{{L}_{\tau }}}{{\rm{Tr}}{{\mathcal{T}}}^{{L}_{\tau }}},\quad {\mathcal{T}}={e}^{-k{{\mathcal{H}}}_{0}/2}{e}^{-k{{\mathcal{H}}}_{1}}{e}^{-k{{\mathcal{H}}}_{0}/2}.$$

$\langle {\mathcal{A}}\rangle$ can be computed as well by averaging *A*_cl_(***S***_*τ*_), evaluated over configurations distributed according to equation (5). (The value of *τ* is arbitrary; hence, one may gain statistics by averaging over *τ*).

Finally, let us emphasize that both ${\mathcal{T}}$ and $\widetilde{{\mathcal{T}}}$ are self-adjoint, positive-definite, transfer matrices that share the crucial symmetries discussed in ‘Crucial symmetries’.

### Observables

The quantities defined in ‘One-time observables’ were aimed at probing the ground state as *k* (and, hence, *Γ* (equation (6))) varies. These quantities will always be averaged over disorder before we proceed with the analysis.

Instead, the time correlations in ‘Two-times observables’ will probe the excitations. These time correlations will be analysed individually for each sample (sample-to-sample fluctuations are considered in ‘Fitting process and estimating the Euclidean correlation length’).

#### One-time observables

We consider the *L*^*D*^ × *L*^*D*^ correlation matrices *M* and $\widehat{M}$ (refs. ^45,46^) with **p** = (2π/*L*, 0) or (0, 2π/*L*):

9 $${M}_{x,y}=\langle {\sigma }_{x}^{Z}{\sigma }_{y}^{Z}\rangle ,\quad {[\widehat{M}]}_{x,y}={M}_{x,y}{e}^{\text{i}{\bf{p}}\cdot (x-y)}.$$

The *n*-body spin-glass susceptibilities at both zero and minimal momentum are

10 $${\chi }^{(n)}=\frac{\overline{{\rm{Tr}}\left[{M}^{n}\right]}}{{L}^{D}},\quad {F}^{(n)}=\frac{1}{{L}^{D}}\overline{{\rm{Tr}}\left[\widehat{M}{M}^{n-1}\right]}.$$

*χ*^(*n*)^ and *F*^(*n*)^ give us access to the second-moment correlation length (see, for example, ref. ^48^):

11 $${\xi }^{(n)}=\frac{1}{2\sin ({\rm{\pi }}/L)}\sqrt{\frac{{\chi }^{(n)}}{{F}^{(n)}}-1}.$$

As *L* grows, *χ*^(*n*)^ and *ξ*^(*n*)^ remain of order 1 in the paramagnetic phase, whereas, in the critical region, they diverge as ${\chi }^{(n)}\propto {L}^{{\gamma }^{(n)}/\nu }$ and *ξ*^(*n*)^ ∝ *L*. In the spin-glass phase, *χ*^(*n*)^ ∝ *L*^*D*(*n*−1)^ (*ξ*^(*n*)^ ∝ *L*^*a*^ with some unknown exponent *a* > 1).

Our *χ*^(*n*=2)^ and *ξ*^(*n*=2)^ are just the standard quantities in the spin-glass literature^66,67^. In fact, in the simplest approximation (see ref. ^47^ for a more paused exposition) at criticality and for large separations *r* between *x* and *y*, *M*_*x*,*y*_ ∝ *v*_*x*_*v*_*y*_/*r*^*a*^ with *v*_*x*_, *v*_*y*_ of order 1 (so, *γ*^(*n*)^/*ν* = (*n* − 1)*D* − *na* in this approximation). Hence, if *D* > *a*, *γ*^(*n*)^ grows with *n*. Indeed, *n* = 3 turns out to be a good compromise between statistical errors, which grow with *n*, and a strong enough critical divergence of *χ*^(*n*)^ (*χ*^(*n*=2)^ barely diverges^5^).

Besides, we computed the Binder cumulant using *Q*_2_ = *L*^*D*^*χ*^(*n*=2)^ as

12 $$B=\frac{{Q}_{4}}{{Q}_{2}^{2}},\quad {Q}_{4}=\sum _{x,y,z,u}\overline{{\langle {\sigma }_{x}^{Z}{\sigma }_{y}^{Z}{\sigma }_{z}^{Z}{\sigma }_{u}^{Z}\rangle }^{2}}.$$

The Gaussian nature of the fluctuations in the paramagnetic phase causes *B* to approach 3 as *L* grows for fixed *k* < *k*_c_. *B* reaches different large-*L* limits for fixed *k* ≥ *k*_c_ (for *k* > *k*_c_, different behaviours are possible, depending on the degree of replica symmetry breaking^68^).

#### Two-times observables

Let us start by defining the time-correlation function of an observable ${\mathcal{A}}$ (for simplicity, consider a product of *σ*^*Z*^ operators at some sites):

13 $${C}_{{\mathcal{A}}}(\tau )=\frac{{\rm{Tr}}\,{\mathcal{A}}\,{{\mathcal{T}}}^{\tau }\,{\mathcal{A}}\,{{\mathcal{T}}}^{{L}_{\tau }-\tau }}{{\rm{Tr}}\,{{\mathcal{T}}}^{{L}_{\tau }}}.$$

${C}_{{\mathcal{A}}}(\tau )$ can be computed from our spin configurations distributed according to the classical weight (equation (5)) by averaging ${\sum }_{{\tau }_{1}=0}^{{L}_{\tau }-1}{A}_{{\rm{cl}}}({{\bf{S}}}_{{\tau }_{1}}){A}_{{\rm{cl}}}({{\bf{S}}}_{{\tau }_{1}+\tau })/{L}_{\tau }^{2}$ (notation as in ‘The Trotter–Suzuki formula’).

Specifically, we have considered

14 $$C(\tau )=\frac{\sum _{x}{C}_{{\sigma }_{x}^{Z}}(\tau )}{{L}_{\tau }^{D}},\quad {Q}_{2}(\tau )=\frac{\sum _{x,y}{C}_{{\sigma }_{x}^{Z}{\sigma }_{y}^{Z}}(\tau )}{{L}_{\tau }^{2D}}.$$

Let us briefly recall some general results^64,65^ for ${C}_{{\mathcal{A}}}(\tau )$ that follow from the spectral decomposition of the transfer matrix (to simplify the notation, let us first disregard the parity symmetry and consider PBCs).

The *τ*-dependence is given by the additive contribution of every pair of states *E*_*n*_ < *E*_*m*_ (*n* = 0 is the ground state). Each pair generates an exponentially decaying term ${B}_{n,m}[{{\rm{e}}}^{-\tau /{\eta }_{n,m}}+{{\rm{e}}}^{-({L}_{\tau }-\tau )/{\eta }_{n,m}}]$ with correlation length *η*_*n*,*m*_ = 1/(*kΔ*_*n*,*m*_), where *Δ*_n,*m*_ = (*E*_*m*_ − *E*_*n*_). The amplitude is ${B}_{n,m}={{\rm{e}}}^{-{L}_{\tau }/{\eta }_{0,n}}\,| \langle n| A| m\rangle {| }^{2}/\widehat{Z}$, with $\widehat{Z}=1+\sum _{n\  > \ 0}{{\rm{e}}}^{-{L}_{\tau }/{\eta }_{0,n}}$. Hence if *L*_*τ*_ ≫ *η*_0,*n*_ the contribution of this pair of states can be neglected. Besides, in the presence of parity symmetry, for even ${\mathcal{A}}$ we find *B*_*n*,*m*_ = 0 if the parity of $\left|n\right\rangle$ and $\left|m\right\rangle$ differ (for odd operators, *B*_*n*,*m*_ = 0 if both parities are equal). This is why the largest correlation length for *Q*_2_(*τ*) is the maximum of *η*_e_ and *η*_o_, whereas the relevant correlation length for *C*(*τ*) is *η* (Extended Data Fig. 1).

**Extended Data Fig. 2:**
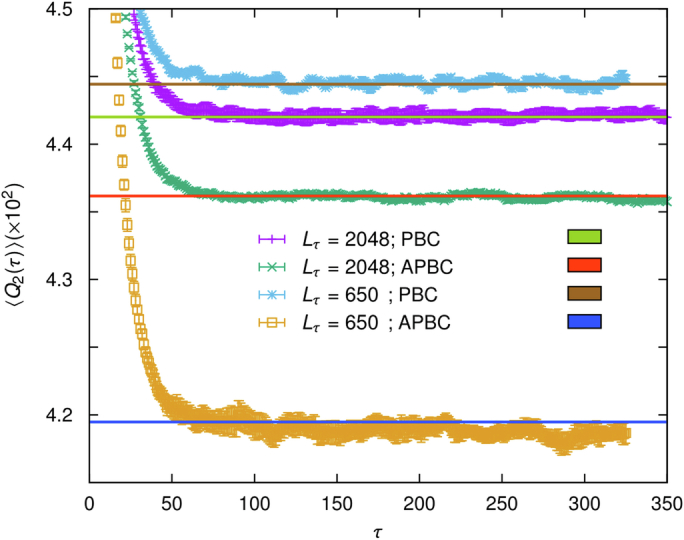
Quantifying the finite-temperature effects. Even correlation functions *Q*_2_(*τ*) defined in Eq. (14), as computed for a single sample of *L* = 20 at *k* = 0.29 ≈ *k*_c_. The corresponding *Q*_2_ value calculated from ${\rm{Tr}}{M}^{2}/{L}^{2D}$ is represented by a complementary colored horizontal line. Points are statistical averages, and errors are one standard deviation. Our data set is fully described in Extended Data Table 1. Source Data

Moreover, for even operators, every state $\left|n\right\rangle$ provides an additive contribution to a *τ*-independent term (namely, the plateau in Extended Data Fig. 2): ${{\rm{e}}}^{-{L}_{\tau }/{\eta }_{0,n}}\,| \langle n| A| n\rangle {| }^{2}/\widehat{Z}$. Instead, for odd operators, $| \langle n| A| n\rangle | =0$ (hence, odd operators lack a plateau). To manage a situation with APBCs, one just needs to add an extra parity operator as a final factor in both the numerator and the denominator of both equations (8) and (13). If parity is a symmetry, as is the case for our problem, $\widehat{Z}$ is modified as $\widehat{Z}=1+\sum _{n > 0}{p}_{n}{{\rm{e}}}^{-{L}_{\tau }/{\eta }_{0,n}}$ (*p*_*n*_ = ±1 is the parity of the state) and the contribution to the APBC plateau gets an extra factor *p*_*n*_, as well.

One may wish to average over samples ${C}_{{\mathcal{A}}}(\tau )$. The dominant time decay for a given sample will be approximately *B*e^−*τ*/*η*^. Hence, the time decay for the averaged $\overline{{C}_{{\mathcal{A}}}(\tau )}$ is an integral ∫d*B*d*ηρ*(*B*, *η*)*B*e^−*τ*/*η*^, where *ρ*(*B*, *η*) is the corresponding probability density (over the samples). For simplicity, let us assume that fluctuations of the amplitude *B* are mild. Then, the scaling in equation (4) implies that, for large *L*_*τ*_, the asymptotic time decay of $\overline{{C}_{{\mathcal{A}}}(\tau )}$ is a function of the scaled time *τ*/*L*^*z*^, where *z* is a dynamic exponent that applies to the parity of *A*. One just needs to change the integration variable as *u* = *η*/*L*^*z*^ and recall the scaling form *ρ*(*η*) ≈ *f*(*η*/*L*^*z*^)/*L*^*z*^, where *f* is a suitable scaling function.

#### The limit of zero temperature

We shall assume that we can reach *L*_*τ*_ large enough so that ${{\rm{e}}}^{-{L}_{\tau }/{\eta }_{{\rm{e}}}},\;{{\rm{e}}}^{-{L}_{\tau }/{\eta }_{{\rm{o}}}}\ll 1$ (the notation is explained in Extended Data Fig. 1). Moreover, we shall not assume that ${\epsilon }\equiv {{\rm{e}}}^{-{L}_{\tau }/\eta }$ is small (in fact, for some samples, one could even have *ϵ* ≈ 1).

Now, consider an even operator ${\mathcal{A}}$, and let us define ${{\mathcal{A}}}_{{\rm{e}}}=\langle {0}_{{\rm{e}}}| {\mathcal{A}}| {0}_{{\rm{e}}}\rangle$ and ${{\mathcal{A}}}_{{\rm{o}}}=\langle {0}_{{\rm{o}}}| {\mathcal{A}}| {0}_{{\rm{o}}}\rangle$ (the thermal expectation value at exactly *T* = 0 is ${{\mathcal{A}}}_{{\rm{e}}}$). The plateau at *τ* ≫ *η*_e_, *η*_o_ (Extended Data Fig. 2) is

15 $${C}_{{\mathcal{A}}}(\tau \gg {\eta }_{{\rm{e}}},{\eta }_{{\rm{o}}})={{\mathcal{A}}}_{{\rm{e}}}^{2}+[{{\mathcal{A}}}_{{\rm{o}}}^{2}-{{\mathcal{A}}}_{{\rm{e}}}^{2}]\frac{\zeta {\epsilon }}{1+\zeta {\epsilon }},$$

where *ζ* = 1 for PBCs and *ζ* = −1 for APBCs. Thus, we get for the plateau of *Q*_2_(*τ*)

16 $${Q}_{2}(\tau \gg {\eta }_{{\rm{e}}},{\eta }_{{\rm{o}}})={Q}_{2,{\rm{e}}}+[{Q}_{2,{\rm{o}}}-{Q}_{2,{\rm{e}}}]\frac{\zeta {\epsilon }}{1+\zeta {\epsilon }},$$

where *Q*_2,e_ and *Q*_2,o_ are, respectively, the average over all pairs (*x*, *y*) of ${{\mathcal{A}}}_{{\rm{e}}}$ and ${{\mathcal{A}}}_{{\rm{o}}}$ (${\mathcal{A}}={\sigma }_{x}^{Z}{\sigma }_{y}^{Z}$; recall equation (14)). Let us give a few hints about the derivation of equations (15) and (16). The contribution of state $\left|n\right\rangle$ to the plateau is ${{\rm{e}}}^{-{L}_{\tau }/{\eta }_{0,n}}\,| \langle n| A| n\rangle {| }^{2}{p}_{n}/\widehat{Z}$ where *p*_*n*_ = 1 for PBCs whereas, for APBCs, *p*_*n*_ = 1 for even states and *p*_*n*_ = −1 for odd states. As explained before, we just keep the states $\left|{0}_{{\rm{e}}}\right\rangle$ and $\left|{0}_{{\rm{o}}}\right\rangle$ when estimating the plateau.

To excellent numerical accuracy, the left-hand side of equation (16) is also the value one gets for ${\rm{Tr}}{M}^{2}/{L}^{2D}$ (Extended Data Fig. 2). In fact, the difference between ${\langle {\mathcal{A}}\rangle }^{2}$ and its plateau is $\zeta {\epsilon }{({{\mathcal{A}}}_{{\rm{e}}}-{{\mathcal{A}}}_{{\rm{o}}})}^{2}/{(1+\zeta {\epsilon })}^{2}$ (hence, quadratic in $({{\mathcal{A}}}_{{\rm{e}}}-{{\mathcal{A}}}_{{\rm{o}}})$ rather than linear, as in equation (15)).

Now, despite their simplicity, two important consequences follow from equations (15) and (16).

First, the limit *T* → 0 (or *L*_*τ*_ → *∞*) is approached monotonically. Furthermore, the systems with PBCs and APBCs (Extended Data Fig. 2) approach the limit from opposite sides. We have explicitly checked all our samples, finding no instance where the APBC plateau lies above the PBC one (it is intuitively natural to expect that the PBC system will be more ordered than the APBC one). Hence, we conclude that the samples with PBCs converge to *T* → 0 from above, whereas the APBC ones converge from below.

Second, as 0 < *Q*_2_(*τ*) < 1 also for APBCs, one has $$0 < {Q}_{2}^{{\rm{APBC}}}(\tau \gg {\eta }_{{\rm{e}}},{\eta }_{{\rm{o}}})$$$$ < {Q}_{2,{\rm{e}}} < 1$$. Thus, $$| {Q}_{2}^{{\rm{APBC}}}(\tau \gg {\eta }_{{\rm{e}}},{\eta }_{{\rm{o}}})-{Q}_{2,{\rm{e}}}|  < 1$$, and we conclude that ∣*Q*_2,o_ − *Q*_2,e_∣ < (1 − *ϵ*)/*ϵ*. Hence, quite paradoxically, the particularly difficult samples with *ϵ* ≈ 1 generate a very small finite-temperature bias in the PBC estimator (compare the *L*_*τ*_ dependence of the PBC and the APBC plateaus in Extended Data Fig. 2). This is why we are able to reach the *T* → 0 limit for the even operators, even if a fraction of our samples suffers from a large value of *ϵ*.

#### Simulation details

We followed two approaches: exact diagonalization of the transfer matrix (equation (8)) and Markov chain Monte Carlo simulations of the classical weight (equation (5)). GPUs were crucial for both. We provide here only the main details (the interested reader is referred to ref. ^69^).

Exact diagonalization is limited to small systems (up to *L* = 6 in our case). Indeed, the transfer matrix has a size ${2}^{{L}^{2}}\times {2}^{{L}^{2}}$. Parity symmetry has allowed us to represent ${\mathcal{T}}$ as a direct sum of two submatrices of half that size^69^. Specifically, we computed the eigenvalues ${{\rm{e}}}^{-k{E}_{0,{\rm{e}}}}$, ${{\rm{e}}}^{-k{E}_{1,{\rm{e}}}}$, ${{\rm{e}}}^{-k{E}_{0,{\rm{o}}}}$ and ${{\rm{e}}}^{-k{E}_{1,{\rm{o}}}}$, as well as the corresponding eigenvectors $| {0}_{{\rm{e}}}\rangle$, $| {0}_{{\rm{o}}}\rangle$, $\left|{1}_{{\rm{e}}}\right\rangle$ and $\left|{1}_{{\rm{o}}}\right\rangle$, for 1,280 samples of *L* = 6 at *k* = 0.31 and 0.305 (the same samples at both *k* values). We repeated the calculations for a subset of 350 samples at *k* = 0.3 and 0.295. We managed to keep the computing time within an acceptable time frame of 20 min per diagonalization using 256 GPUs, thanks to a highly tuned custom matrix-vector product^69^. These computations have proven to be invaluable in the process of taking the limit *L*_*τ*_ → *∞* (‘More about exact diagonalization’ in Methods).

Our Monte Carlo simulations used the parallel tempering algorithm^70^, implemented over the *k* parameter in equation (5), to ensure equilibration. We equilibrated 1,280 samples for each lattice size (Extended Data Table 1). As a rule, we estimated errors using the bootstrap method^71^, as applied to the disordered average.

We simulated six real replicas of every sample (six statistically independent simulations of the system), for several reasons. Replicas allowed us to implement the equilibration tests based on the tempering dynamics^56^. They also provided unbiased estimators of products of thermal averages (equation (10)). Finally, fluctuations between replicas allowed us to estimate errors for the time-correlation functions (equation (14)), as computed in a single sample (‘Fitting process and estimating the Euclidean correlation length’).

The Monte Carlo code exploits a three-level parallelization (multispin coding, domain decomposition and parallel tempering), which kept the spin-update time below 0.5 ps (ref. ^69^), competitive with dedicated hardware^22^.

### More about exact diagonalization

**Extended Data Fig. 3:**
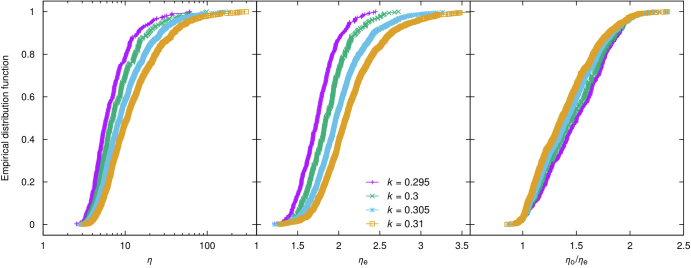
Sample dependence of the Euclidean correlation lengths. Empirical distribution function of the different Euclidean correlation lengths presented in the system, for different values of *k*. Data from the exact diagonalization of a *L* = 6 system. Data from *k* = 0.295, and *k* = 0.3 are calculated over 320 samples, instead of 1280. Source Data

The schematic representation of the spectrum in Extended Data Fig. 1 is based on the distribution functions in Extended Data Fig. 3 (we typically compute the inverse distribution function; see ‘Fitting process and estimating the Euclidean correlation length’ for details).

Indeed, the correlation length *η* displays very large sample-to-sample fluctuations (to the point that a logarithmic representation is advisable) and a very strong *k*-dependence (Extended Data Fig. 3, left). In contrast, *η*_e_ is always a number of order one in our *L* = 6 samples (Extended Data Fig. 3, middle). Furthermore, *η*_o_/*η*_e_ ≈ 1 in all cases (Extended Data Fig. 3, right).

**Extended Data Fig. 4:**
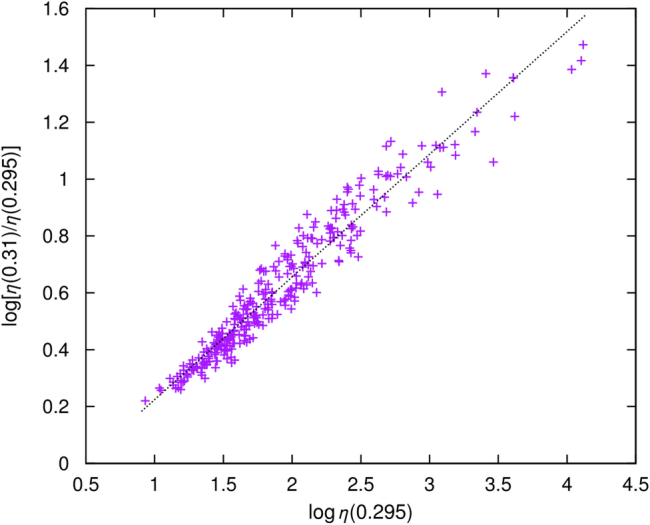
Dependence on *k* of the odd correlation length *η.* The figure shows that the logarithm of the ratio of *η*(*k* = 0.31) and *η*(*k* = 0.295) (computed for the same *L* = 6 sample through exact diagonalization) is very approximately a linear function of $\log \eta (k=0.295)$, with a positive slope. The figure shows data for the 350 samples that we have studied at both *k* = 0.295 and *k* = 0.31. Source Data

In fact, the distribution for *η* is a Lévy flight (Fig. 4c; for large *η*, $F(\eta )=1-B/{\eta }^{b}$). The mechanism allowing exponent *b* to vary with *k* (hence, with transverse field (equation (6))) is sketched in Extended Data Fig. 4. Let us compare the value of *η* for the same sample at *k*_1_ and *k*_2_ (*k*_1_ < *k*_2_). With great accuracy, $\eta ({k}_{2})=\alpha {[\eta ({k}_{1})]}^{1+\beta }$, where *α* and *β* are constants (for fixed *k*_1_ and *k*_2_) and *β* > 0. Thus, ordering samples according to their *η*(*k*_1_) is the same as ordering by *η*(*k*_2_), because one is a monotonically increasing function of the other. Hence, the same sample occupies percentile *F* in the distribution for both *k*_1_ and *k*_2_. It follows that *b*(*k*_2_) = *b*(*k*_1_)/(1 + *β*) for the exponent characterizing the Lévy flight. In other words, because *b*(*k*_2_) < *b*(*k*_1_), the tail at large *η* becomes heavier as *k* increases (see ‘On the magnetic susceptibilities’ for an extended discussion).

### The critical point and critical exponents

After taking care of the *L*_*τ*_ → *∞* limit (within errors) in our study of the phase transition, we still need to cope with the finite spatial dimension *L*. We shall do so using finite-size scaling^72–75^ (Fig. 1c). The main questions we shall address are the computation of the critical exponents and the estimation of the critical point. Our main tool will be the quotients method^48,60,76^, which, surprisingly, keeps our two questions somewhat separate.

The quotients method starts by comparing a dimensionless quantity at two sizes *L*_*a*_ < *L*_*b*_ (in our case, *ξ*^(3)^/*L* as a function of *k*). First, we located a coupling k^*^(*L*_*a*_, *L*_*b*_) such that the curves for *L*_*a*_ and *L*_*b*_ cross (Fig. 1b). Now, for dimensionful quantities *A*, scaling in the thermodynamic limit as ${\xi }^{{x}_{A}/\nu }$, we consider the quotient ${Q}_{A}={A}_{{L}_{a}}/{A}_{{L}_{b}}$ at k^*^(*L*_*a*_, *L*_*b*_). Barring scaling corrections, ${Q}_{A}={({L}_{a}/{L}_{b})}^{{x}_{A}/\nu }$, which yields an effective estimate of *x*_*A*_/*ν*. Indeed, considering only the leading correction to the scaling exponent *ω*, we have for the effective exponent:

17 $${\left.\frac{{x}_{A}}{\nu }\right|}_{{L}_{a},{L}_{b}}=\frac{{x}_{A}}{\nu }\,+\,\frac{1}{\log ({L}_{b}/{L}_{a})}\log \frac{1+{D}_{A}{L}_{b}^{-\omega }}{1+{D}_{A}{L}_{a}^{-\omega }},$$

where *D*_*A*_ is an amplitude. Our estimates for the effective exponents can be found in Extended Data Table 2. Yet, effective exponents need to be extrapolated to the thermodynamic limit through equation (17). Unfortunately, we have not been able to estimate exponent *ω*, as there were two difficulties. First, the range of *L* values at our disposal was small. Second the analytic background^48^ for the *r* = 2 observables and for the Binder parameter (‘One-time observables’) compete with the *L*^−*ω*^ corrections. Hence, we followed an alternative strategy. We fitted our effective exponents to equation (17) with fixed *ω* (the fitting parameters were the extrapolated *x*_*A*_/*ν* and the amplitude *D*_*A*_). To account for our ignorance about *ω*, we made it vary in a wide range 0.5 ≤ *ω* ≤ 2. The central values in equations (2) and (3) were obtained with *ω* = 1, whereas the second error estimate accounts for the *ω*-dependence of *x*_*A*_/*ν*. Indeed, the first error estimate is the statistical error as computed for *ω* = 1, whereas the second error estimate is the semi-difference between the extrapolations to infinite size obtained with *ω* = 2.0 and *ω* = 0.5. To take into account the data correlation, we employed a bootstrap method^77^. We considered only the diagonal part of the covariance matrix in the fits and performed a new fit for every bootstrap realization. Errors were computed from the fluctuations of the fitting parameters. Fortunately, the systematic errors turned out to be comparable (for 1/*ν* smaller) with the statistical ones.

Like the critical point, we expected scaling corrections of the form k*(*L*_*a*_, *L*_*b*_) = *k*_c_ + *D*_*k*_*F*(*L*_*a*_, *L*_*b*_), where *D*_*k*_ is an amplitude^78^:

18 $$F({L}_{a},{L}_{b})={L}_{a}^{-\left(\omega +\frac{1}{\nu }\right)}\frac{1-{s}^{-\omega }}{{s}^{1/\nu }-1},\quad s=\frac{{L}_{b}}{{L}_{a}}\,.$$

**Extended Data Fig. 5:**
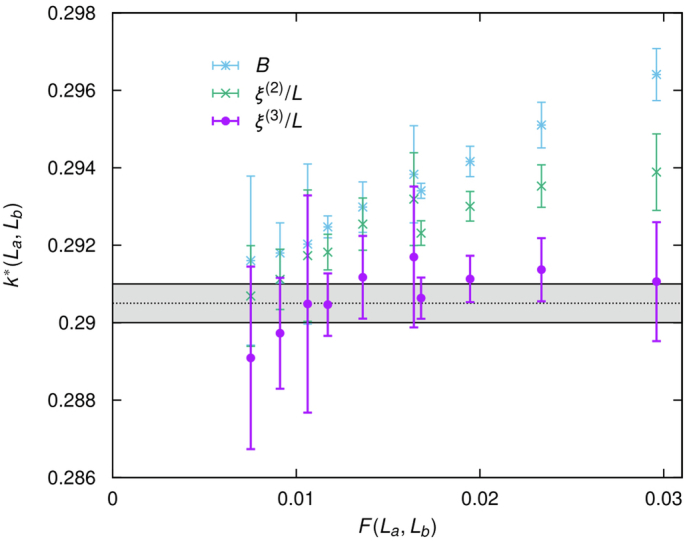
Determination of the critical point. When studied as a function of *k* on two system sizes *L*_*a*_ < *L*_*b*_, the curves for dimensionless quantities cross at a point k*(*L*_*a*_, *L*_*b*_), see Fig. 1-b. The figure shows k* (as computed for *B*, *ξ*^(2)^/*L* and *ξ*^(3)^/*L*) versus *F*(*L*_*a*_, *L*_*b*_) (18). We set 1/*ν* = 0.7 and *ω* = 1 to compute *F*(*L*_*a*_, *L*_*b*_). The curves should extrapolate linearly to *k*_c_ as *F*(*L*_*a*_, *L*_*b*_) tends to zero. The shaded area encompass our uncertainty in the estimation of *k*_c_. Points are statistical averages, and errors are one standard deviation. Our data set is fully described in Extended Data Table 1. Source Data

Unfortunately, this result is not of much use without a *ω* estimate. Fortunately, see Extended Data Table 2 and Extended Data Fig. 5, the values of k*(*L*_*a*_, *L*_*b*_) obtained from *ξ*^(3)^/*L* seem not to depend on size. In fact, our estimate for *k*_c_ in equation (2) is an interval that encompasses all our results (the shaded area in Extended Data Fig. 5). Furthermore, the crossing points for *B* and *ξ*^(2)^/*L* (Extended Data Fig. 5) seem also reasonably well represented by equation (18).

### Fitting process and estimating the Euclidean correlation length

Our aim in this section is to determine the relevant correlation lengths for *C*(*τ*) and *Q*_2_(*τ*) at a fixed *k*, for our *N*_S_ = 1,280 samples. The results are characterized through their empirical distribution function (Figs. 3 and 4). Given that *N*_S_ is large, we needed an automated approach.

The first step was estimating, for a given sample, *C*(*τ*) and *Q*_2_(*τ*), as well as their standard errors, by using our six replicas. Now, the analysis of a noisy correlation function (such as *C*(*τ*) and *Q*_2_(*τ*); see, for example, Extended Data Fig. 2) needs a fitting window^79,80^. We chose the window upper limit as ${\tau }_{{\rm{w}},f}\equiv \mathop{\min }\limits_{\tau }\{\tau | \,f(\tau )=3.5{\sigma }_{f(\tau )}\}$, with *f*(*τ*) either *C*(*τ*) or *Q*_2,s_(*τ*) = *Q*_2_(*τ*) − *Q*_2,pl_, where *Q*_2,pl_ is the plateau (Extended Data Fig. 2) and *σ*_*f*(*τ*)_ is the corresponding standard error. We faced two problems. First, for odd *C*(*τ*), some samples have *τ*_w,*C*_ ≥ *L*_*τ*_/2. For these samples, *η* > *L*_*τ*_, and hence, it was impossible to estimate them (Fig. 4b). *Q*_2,s_(*τ*) was not affected by this problem (Fig. 3). Second, we needed to estimate the plateau *Q*_2,pl_. To do so, we fitted *Q*_2_(*τ*) for *τ* ∈ [*L*_*τ*_/4, *L*_*τ*_/2] to a constant *Q*_2,pl_. In the few exceptions where this fit was not acceptable (as determined by its figure of merit *χ*^2^/degrees of freedom (dof) computed with the diagonal part of the covariance matrix), we proceeded as explained below (we used ${\tau }_{{\rm{w}},{Q}_{2}}={L}_{\tau }/2$ in those cases).

We determined the correlation lengths through fits to $$C(\tau )\,=$$$$B[{\text{e}}^{-\tau /\eta }+{\text{e}}^{(\tau -{L}_{\tau })/\eta }]$$ and $${Q}_{2}(\tau )={Q}_{2,\text{pl}}+{B}_{\text{e}}{e}^{-\tau /{\eta }_{\text{e}}}+{B}_{\text{o}}{e}^{-\tau /{\eta }_{\text{o}}}$$. The fitting parameters were the amplitudes and the correlation lengths (and, for the above-mentioned exceptional samples, also *Q*_2,pl_). Actually, for *Q*_2_(*τ*) we considered fits with one and with two exponential terms, keeping the fit with the smallest *χ*^2^/dof, as we could not determine which of the two correlation lengths obtained in the fit corresponded to the even gap (Extended Data Fig. 1). Hereafter, *η*_e_ is the larger of the two. As for the lowest limit of the fitting window, we started from $${\tau }_{\min ,{Q}_{2}}=1$$ and $${\tau }_{\min ,C}={\tau }_{{\rm{w}},C}/10$$, and we kept increasing the corresponding $${\tau }_{\min }$$ until *χ*^2^/dof went below 0.5 for *Q*_2_ (below 1 for *C*(*τ*)).

Finally, we determined the empirical distribution function for the correlation lengths. Let *X* be either $\log \eta$ or *η*_e_ (see below for some subtleties regarding *η*). We actually computed the inverse function *X*[*F*] by sorting in increasing order the *N*_S_ values of *X* and setting $X[F=i/{N}_{{\rm{S}}}]$ as the *i*th item in the ordered list. We obtained *X*[*F*] at the value of *k* of interest through linear interpolation of *X*[*F*] computed at the two nearest values of *k* in the parallel tempering grid. To estimate the errors in *X*[*F*], we employed a bootstrap method with 10,000 as the resampling value. In each resampling, we randomly picked *N*_S_ values of *X*. For the chosen sample, we extracted *X* from a normal distribution centred in *X* as obtained from the fit. The standard deviation was the fitting error for *X*.

For $X=\log \eta$, we needed to cope with the problem that we could determine *X* for only *N*_OK_ of our *N*_S_ samples. We decided to determine *X*[*F*] only up to ${F}_{{\rm{safe}}}\equiv ({N}_{{\rm{OK}}}-4\sqrt{({N}_{{\rm{S}}}-{N}_{{\rm{OK}}}){N}_{{\rm{OK}}}/{N}_{{\rm{S}}}})/{N}_{{\rm{S}}}$ (the maximum possible *F* minus four standard deviations). We imposed for every bootstrap resampling that *X* could be obtained in at least *F*_safe_*N*_S_ samples (this limitation was irrelevant in practice).

Let us conclude by mentioning that the estimates in equation (4) were obtained through a joint fit for *η*_e_[*F*], with *F* = 0.5, 0.6, 0.7, 0.8 or 0.9. Errors were estimated as explained in ‘The critical point and critical exponents’.

### On the magnetic susceptibilities

**Extended Data Fig. 6:**
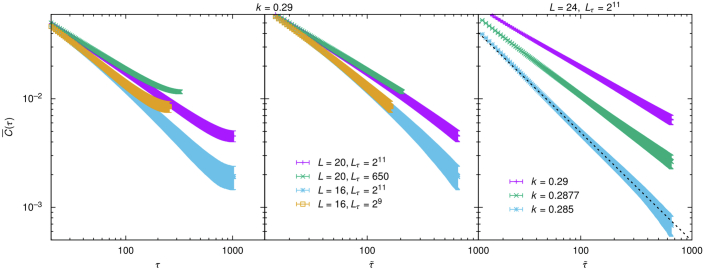
The power-law decay with Euclidean time of the odd correlation functions. Sample-averaged Euclidean correlation function as a function of the Euclidean distance *τ* (**left** panel), $\widetilde{\tau }=\frac{{L}_{\tau }}{{\rm{\pi }}}\sin ({\rm{\pi }}\tau /{L}_{\tau })$ to avoid distortions due to the periodic boundary conditions (**center** and **right** panels). **Left** and **center** panels show the system size dependence for *k* = 0.29, despite the **right** panel shows the *k*-dependence for the bigger system, *L* = 24 and *L*_*τ*_ = 2^11^. The dashed line in the **right** panel is a guide to the eye to show the critical exponent $\widetilde{b}=1$ encountered for *k* = 0.285 (see Sect. 5.6). Points are statistical averages, and errors are one standard deviation. Our data set is fully described in Extended Data Table 1. Source Data

The sample-averaged linear susceptibility to an external magnetic field at *T* = 0, ${\chi }_{{\rm{lin}}}^{(h)}$, can diverge only if $\overline{C(\tau )}$ decays slowly for large *τ* (because ${\chi }_{{\rm{lin}}}^{(h)}=1+2{\sum }_{\tau =1}^{\infty }\overline{C(\tau )}$; Extended Data Fig. 6). Yet, the periodicity induced by the PBCs (Extended Data Fig. 6a) made it difficult to study the behaviour at large *τ*. Fortunately, representing $\overline{C(\tau )}$ as a function of $\widetilde{\tau }=\frac{{L}_{\tau }}{{\rm{\pi }}}\sin ({\rm{\pi }}\tau /{L}_{\tau })=\tau [1+{\mathcal{O}}({\tau }^{2}/{L}_{\tau }^{2})]$ greatly alleviated this problem (Extended Data Fig. 6b). Thus armed, we could study the long-time decay of $C(\tau )\propto 1/{\widetilde{\tau }}^{\widetilde{b}}$ as a function of *k* (Extended Data Fig. 6c). Indeed, $\widetilde{b}$ decreased as *k* increased. As *C*(*τ*) ≈ *B*e^−*τ*/*η*^ for any sample, the mechanism discussed in ‘More about exact diagonalization’ in Methods is clearly at play. The heavy tail of *F*(*η*) became heavier as *k* increased, which resulted in a decreasing exponent $\widetilde{b}$. In fact, the critical exponent $\widetilde{b}=1$ was encountered at *k* ≈ 0.285, well into the paramagnetic phase (${\chi }_{{\rm{lin}}}^{(h)}=\infty$ if $\widetilde{b}\le 1$).

The Lévy-flight perspective provides a simple explanation for the results in refs. ^6,41^. In a single sample, the different susceptibilities to a magnetic field (linear, third-order and so on) are proportional to increasing powers of *η*. Hence, the existence of the disorder average of a given (generalized) susceptibility boils down to the existence of the corresponding moment of the distribution *F*(*η*). As soon as *F*(*η*) decays for large *η* as a power law, some disorder-averaged susceptibility will diverge (probably a higher-order one). Lower-order susceptibilities diverge at larger values of *k*. Hence, it is not advisable to use this approach to locate the critical point.

## Online content

Any methods, additional references, Nature Portfolio reporting summaries, source data, extended data, supplementary information, acknowledgements, peer review information; details of author contributions and competing interests; and statements of data and code availability are available at 10.1038/s41586-024-07647-y.

### Source data

Source Data Fig. 1

Source Data Fig. 2

Source Data Fig. 3

Source Data Fig. 4

Source Data Extended Data Fig. 2

Source Data Extended Data Fig. 3

Source Data Extended Data Fig. 4

Source Data Extended Data Fig. 5

Source Data Extended Data Fig. 6

## Figures and Tables

**Extended Data Table 1:**
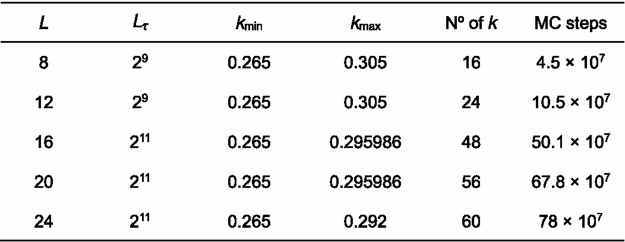
Simulation parameters for the different system sizes

The *k* ranges have been chosen to ensure the critical point *k*_c_ belonged to the range [see Eq (2)]. The *N*° values of *k*, ${k}_{\min }\le k\le {k}_{\max }$, are uniformly distributed. We also provide the number of Metropolis sweeps performed (an elementary step consisted of 30 full-lattice Metropolis sweeps, followed by a Parallel Tempering attempt of exchanging the *k* value).

**Extended Data Table 2:**
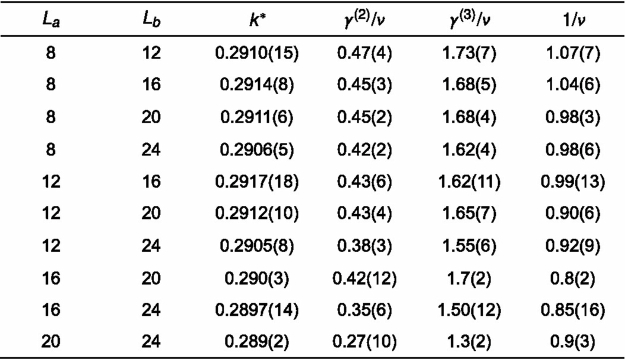
Size-dependent estimates for the critical point and the critical exponents

Crossing points k*(*L*_*a*_, *L*_*b*_) obtained for *ξ*^(3)^/*L* and the size-dependent, effective critical exponents [see Eq. (17)] as estimated from *χ*^(2)^ (*γ*^(2)^/*ν*), *χ*^(3)^ (*γ*^(3)^/*ν*) and ∂_*k*_*ξ*^(3)^/*L* (1/*ν*). Errors indicate one standard deviation and have been computed using a bootstrap method. Our data set is fully described in Extended Data Table 1.

## Data Availability

The data can be obtained from the corresponding author (I.G.-A.P.) upon request. Source data are provided with this paper.

## Code availability

The authors have made their code publicly available in ref. ^69^.
